# A Rare Case: Is It Hemodialysis Encephalopathy, Dialysis Anxiety, or Both?

**DOI:** 10.7759/cureus.67212

**Published:** 2024-08-19

**Authors:** Vichika Than, Daniel I Casal, Angelo Sica

**Affiliations:** 1 Internal Medicine, St. George's University School of Medicine, True Blue, GRD; 2 Psychiatry and Behavioral Sciences, St. Joseph’s Medical Center, Paterson, USA; 3 Psychiatry/Neurology, Rowan-Virtua School of Osteopathic Medicine, Stratford, USA

**Keywords:** ckd, depression, mood disorders, anxiety, seizures, hemodialysis encephalopathy

## Abstract

We present a case of a 59-year-old female who presented with progressively worsening altered mental status, seizures, and neuropsychiatric symptoms. Over the course of her emergency visit and admission to the hospital, laboratory tests failed to find an offending agent to her presentation. Her clinical presentations supported the diagnosis of encephalopathy, but the actual underlying cause was not found. After careful exclusion of bacterial, viral, and other types of encephalopathy, hemodialysis encephalopathy was a possible diagnosis. The presentation and symptoms of our patient led to a wide range of differentials, and a high index of suspicion was needed throughout her admission in order to obtain the appropriate tests. Computed tomography head (CTH) and electroencephalogram (EEG) were performed and showed results that supported our diagnosis of hemodialysis encephalopathy. Despite the supportive testing results of the brain, there are still some neuropsychiatric symptoms of our patient that remain unexplained. This led us to account for the physical exam, clinical judgment, and the process of elimination to diagnose our patient with anxiety due to dialysis concurrent with hemodialysis encephalopathy. Despite little evidence in the literature supporting the presence of anxiety disorders in patients receiving dialysis, our patient showed alleviated clinical presentation after being prescribed an anti-anxious medication, making this presentation uncommon. In this rare case, we present a patient with possible comorbidity of both hemodialysis encephalopathy and dialysis anxiety that the result from EEG and other tests failed to explain all the symptoms our patient experienced.

## Introduction

Dialysis encephalopathy (DE), also known as “dialysis dementia,” is a well-established clinical syndrome that occurs in patients with chronic kidney disease (CKD) and undergoing long-term hemodialysis. This neuropsychiatric syndrome is characterized by disturbances in speech, cognition, and movement and presents with psychiatric symptoms such as altered affect [1]. Symptom severity fluctuates throughout the early course of the disease. Most symptoms including speech impairment tend to be more noticeable immediately after the dialysis, and speech difficulties seem to be the most consistent manifestation of this syndrome [2]. The more notable presentations of speech impairment include dysarthria and stuttering and can progress to mutism where a patient is no longer able to communicate [3]. Motor disturbances associated with DE include tremors, myoclonus, asterixis, athetosis rigidity, weakness, gait abnormalities, and even seizures [1]. Seizures appear to appear later in the course of the disorder, and according to the literature, myoclonus is reportedly the most common movement disturbance in patients with DE [4]. The cognitive aspect of DE is confusion, memory impairment, disorientation, and bizarre behavior. Another manifestation of the syndrome is behavioral disturbances, which consist of variable affect, paranoia, delusions, and visual and auditory hallucinations [1].

Aluminum toxicity has been a known marker that has been elevated in patients with this DE, but other etiologies cannot be excluded. Aluminum accumulation in dialysis patients is mainly due to high volumes of contaminated dialysate [5]. Poor kidney excretion further contributes to the excess aluminum concentration [5]. Along with the clinical features of DE, an abnormal electroencephalogram (EEG) is important to establish a diagnosis. Typical EEG changes demonstrate increased low-voltage theta waves and anteriorly predominant high-voltage delta wave bursts with a mild slowing of the dominant rhythm [6]. The EEG changes can be contrasted with other causes of encephalopathy, such as metabolic and uremic encephalopathy, due to a relatively normal background frequency. In addition, EEG changes may precede months after clinical manifestations may appear [6]. Treatment involves mostly symptom management. Diazepam has shown promise in managing seizures and even possibly reversing some of the symptoms associated with DE, but researchers have shown that it becomes less effective as the course of the illness progresses [7]. 

Neuropsychiatric conditions including anxiety, depression, cognitive impairment, and somatoform disorders are prevalent in patients with CKD. Cognitive impairment has been associated with CKD, especially in hemodialysis patients [8]. This may be due to the accumulation of uremic toxins, and despite dialysis treatment, cognitive impairment may still persist, indicating that other factors may be involved in the mechanism of cognitive impairment [9]. The prevalence of depression is higher in patients on dialysis and is frequently reported in CKD patients [10]. Depression in CKD patients may be correlated with poor outcomes such as a decline in kidney function, hospitalization, and increased mortality. Studies show that CKD patients with depressive symptoms not only had a greater decline in glomerular filtration rate (GFR) but also are more likely to progress to end-stage renal disease (ESRD) or even death [11]. Depression at the time of dialysis treatment is correlated with lower survival rates, increased frequency of dialysis withdrawal, and increased risk of prolonged hospitalization stay [12]. 

Furthermore, anxiety is another known psychiatric condition in patients with CKD. Studies have shown that the frequency of anxiety was found to be increased in patients with CKD [13]. Just like depression, anxiety symptoms may also be associated with poorer clinical outcomes and increased mortality in CKD patients. Undergoing dialysis and the nature of CKD may trigger somatoform-like disorders where the patient may associate the pain “stemming from the head.” According to the Diagnostic and Statistical Manual of Mental Disorders (DSM-V), somatic symptom disorder (SSD) involves one or more physical symptoms accompanied by a large amount of time, energy, emotion, or behavior-related symptoms causing significant distress in which the physical symptoms may or may not be explained by a medical condition [14]. Studies have shown that anxiety or somatic presentations of psychiatric disorders can occur in adolescent patients with renal failure [15]. Somatic symptoms tend to be associated in patients with mood disorders. Persistent somatoform syndromes are of psychological origin and can cause patients to act out [15]. Therefore, our study aims to increase awareness of a rare clinical condition with overlapping medical and neuropsychiatric symptoms.

## Case presentation

A 59-year-old female with a past medical history of ESRD (requires dialysis three times per week, MWF), peripheral neuropathy, and hypertension presented to the emergency department for evaluation of slurred speech, shakiness, and unsteady gait for the past day. The patient normally takes gabapentin for peripheral neuropathy, but she stopped taking it one week ago. The patient also reported having difficulty hearing from her ears over the past few days. The patient denied any facial droop, numbness, tingling, headache, nausea, or vomiting. 

In the emergency department, the patient was observed to have constant myoclonic-like jerking in both upper extremities that extended downward. Initial blood work was found to have incorrect electrolyte abnormalities suspected to be the result of blood being drawn from the same site/time as calcium gluconate infusion. A computed tomography scan of the head (CTH) was done and showed volume loss with a middle chronic microangiopathic disease. Arterial blood gas showed primary respiratory acidosis. 

Later on during admission, the patient was reported to have seizure-like activities once again that lasted for 30 seconds. The patient was seen and examined by a neurologist who started her on Keppra 500 mg BID for seizure prophylaxis. The patient was transferred to the intensive care unit (ICU) for close airway monitoring. Another arterial blood gas was performed and showed the patient was no longer hypercapnic and oxygenation was well with a normal pH. The patient was then transferred to the medical intensive care unit (MICU) with no indication of emergent intubation at that time. 

During the course of one of the patients' dialysis treatments in the hospital, the patient attempted to get out of bed, requesting for the dialysis to stop, and said that she did not feel well. The patient cried and expressed that she was going to die. She continued saying that she did not know why the dialysis was making her feel like this. The dialysis was stopped, blood was returned to the patient, and the patient admitted to feeling better afterward. The neurology team suspected encephalopathic episodes during dialysis and ordered a video electroencephalogram (EEG) to be performed during the dialysis session. The EEG showed an abnormal 23-hour extended EEG, indicative of a mild diffuse encephalopathy. No epileptiform discharges were noted. One hour after another hemodialysis session, the patient became agitated, anxious, and restless, moving constantly during the session, and the dialysis was stopped. The patient expressed that she felt as if she would lose consciousness and began to cry again. Psychiatry was consulted for anxious distress with physical, conversion-like manifestations only encountered during hemodialysis sessions during this hospitalization.

During the psychiatry encounter, the patient was alert and oriented, able to state the reason for her hospitalization and treatments received, and noted to not have any seizure-like activity or motor dysfunction. However, she was preoccupied with thoughts revolving around dialysis. She endorsed overwhelming anxious thoughts during hemodialysis with anticipatory fears of financial stability if she lost her job. The patient had a prior history of peritoneal dialysis infection resulting in challenges maintaining fluid status and compliance. Additional somatic preoccupation results in physiologic distress akin to anxiety symptoms. A conversion and anxiety-based presentation most likely given stress was endorsed. The patient was prescribed Klonopin 0.5 mg PO BID prn for her anxiety, which she took prior to the start of her next hemodialysis session, and was able to stay calm and finish the session successfully.

## Discussion

There is supporting evidence indicating that DE is a distinct syndrome characterized by clinical and distinct EEG features. Although aluminum is the most known toxin implicated in the syndrome, other etiologies cannot be excluded. DE is characterized by dysarthria, dysphagia, dementia, and motor disturbances with intact consciousness [1]. Our patient presented with several clinical manifestations including gait abnormalities, speech disturbances, and myoclonic jerking of the upper extremities. The patient also had seizures after dialysis treatment. CTH revealed white matter abnormalities and atrophy consistent with encephalopathy. Further EEG abnormalities helped support the diagnosis. The patient also had psychiatric manifestations during the course of hospitalization. Somatoform symptoms such as agitation, anxiousness, tachycardia, shortness of breath, and fear of social maintenance and finances also were present. Given the variety of symptoms that the patient presented with, it is difficult to confirm an exact diagnosis or if there is a neuropsychiatric comorbidity in patients with CKD that is best able to explain the clinical picture.

The association between the brain and kidneys is complex and can further support the neuropsychiatric link in patients with CKD. Neuropsychiatric symptoms are associated with renal dysfunction and progressively affect the patients' quality of life during the course of the disease. Symptom presentations vary depending on the course of the illness, making it more difficult to diagnose and treat [16]. Studies have shown that patients with CKD have a higher prevalence of being hospitalized for psychiatric disorders including depression, dementia, anxiety, and substance abuse compared to other chronic diseases [16]. Depression is a notable symptom present in most patients with ESRD. A study done among dialysis patients revealed that those on peritoneal dialysis had a lower prevalence of depression, anxiety, and sleep disturbances compared to those on hemodialysis. Our patient had both types of dialysis done, and despite her preference for peritoneal dialysis, external factors prevented her from continuing this route and sticking with normal hemodialysis [17]. The history of trauma from previous peritoneal dialysis infection can also play a role in the patient's psychiatric presentation.

Anxiety is another frequent diagnosis present in patients with chronic medical conditions, but there is little evidence in the literature supporting the presence of anxiety disorders in patients with CKD [18]. A previous study noted that patients on dialysis with an anxiety disorder diagnosis who did not receive treatment initially had persisting anxiety symptoms at the 16-month follow-up [19]. In addition, another study has shown that patients on hemodialysis have more frequent anxiety symptoms compared to patients on peritoneal dialysis [17]. Our patient was anxious during the hospitalization, and external factors may have contributed to this clinical picture. Her constant worry about finances, employment, and previous trauma may have caused her to act out and refuse dialysis treatment. These symptoms can be best explained as a type of somatoform disorder where constant worrying and anxiousness have led the patient to present with somatic symptoms of panic disorder. This may have influenced the patient to behave the way she did and be physiologically distressed. Despite the psychiatric presentation, it is difficult to say if her psychiatric presentation was due to DE or stress may have caused her to have presented with a type of somatoform disorder secondary to dialysis.

## Conclusions

DE is a neuropsychiatric condition where a patient presents with disturbances in cognition, movement, speech, and psychiatric presentation. Psychiatric conditions are known to be comorbid with other medical conditions, such as CKD. Clinicians rely heavily on their clinical knowledge, judgment, and intuition alongside tests to guide patient care. At times, this becomes difficult as no objective tests exist with 100% specificity. When objective tests fail to aid in the diagnosis that explains all or the majority of symptoms that the patient experiences, a clinician can diagnose a patient by ruling out other causes and incorporating psychiatric aspects of the disease. In this patient’s case, standard lab tests, CTH, and EEG could explain only a portion of the patient's symptoms, which prompted the recognition of a psychiatric diagnosis of anxiety due to dialysis concurrently with hemodialysis encephalopathy. Our case strongly emphasizes the importance of understanding that there are possible comorbid medical and neuropsychiatric symptoms when diagnosing and explaining a patient's condition. Therefore, complex cases such as our patient prompt physicians to take broader history and order appropriate diagnostics when providing care for their patients. 
